# Measurement of Conditional Relatedness Between Genes Using Fully Convolutional Neural Network

**DOI:** 10.3389/fgene.2019.01009

**Published:** 2019-10-22

**Authors:** Yan Wang, Shuangquan Zhang, Lili Yang, Sen Yang, Yuan Tian, Qin Ma

**Affiliations:** ^1^Key Laboratory of Symbol Computation and Knowledge Engineering of Ministry of Education, College of Computer Science and Technology, Jilin University, Changchun, China; ^2^Department of Obstetrics, The First Hospital of Jilin University, Changchun, China; ^3^School of Artificial Intelligence, Jilin University, Changchun, China; ^4^Department of Biomedical Informatics, College of Medicine, The Ohio State University, Columbus, OH, United States

**Keywords:** conditional relatedness between genes, fully convolutional neural network, co-expression similarity, prior-knowledge similarity, gene network

## Abstract

Measuring conditional relatedness, the degree of relation between a pair of genes in a certain condition, is a basic but difficult task in bioinformatics, as traditional co-expression analysis methods rely on co-expression similarities, well known with high false positive rate. Complement with prior-knowledge similarities is a feasible way to tackle the problem. However, classical combination machine learning algorithms fail in detection and application of the complex mapping relations between similarities and conditional relatedness, so a powerful predictive model will have enormous benefit for measuring this kind of complex mapping relations. To this need, we propose a novel deep learning model of convolutional neural network with a fully connected first layer, named fully convolutional neural network (FCNN), to measure conditional relatedness between genes using both co-expression and prior-knowledge similarities. The results on validation and test datasets show FCNN model yields an average 3.0% and 2.7% higher accuracy values for identifying gene–gene interactions collected from the COXPRESdb, KEGG, and TRRUST databases, and a benchmark dataset of Xiao-Yong et al. research, by grid-search 10-fold cross validation, respectively. In order to estimate the FCNN model, we conduct a further verification on the GeneFriends and DIP datasets, and the FCNN model obtains an average of 1.8% and 7.6% higher accuracy, respectively. Then the FCNN model is applied to construct cancer gene networks, and also calls more practical results than other compared models and methods. A website of the FCNN model and relevant datasets can be accessed from https://bmbl.bmi.osumc.edu/FCNN.

## Introduction

Conditional relatedness between a pair of genes is a degree of the relation between two genes in a certain condition, *e.g.* in cancer tissues or inflammation, implying the probability of these genes jointly involved in a biological process under such cell environment (Wang et al., 2019). It is different from gene–gene interaction meaning a 0/1 (non-interacting/interacting) binary relation between a pair of genes. Measuring such relatedness is a basic tool for understanding the biological and functional relations between genes in a real cell environment (Jelier et al., 2005; Mistry and Pavlidis, 2008). And the measured relatedness is classically used as weights on connections of genes for construction of gene networks in different environments for further biological analysis (Amrine et al., 2015; Li et al., 2018).

Traditionally, expression similarity as well as co-expression is used to measuring conditional relatedness, including Pearson correlation coefficient (PCC) (Eisen et al., 1998), Spearman rank correlation (SRC) (Kumari et al., 2012), mutual information (MI) (Song et al., 2012), partial Pearson correlation (PPC) (Baruch and Albert-László, 2013), and conditional mutual information (CMI) (Kim et al., 2010). PCC can express the linear relationship between a pair of genes, SRC and MI represent the nonlinear relationship, and PPC and CMI indicate the direct linear relationship and the direct nonlinear relationship under the condition of excluding other genes’ interferences, respectively. Expression similarities have been successfully applied in measuring conditional relatedness for constructing gene networks, on which Poliakov et al. identify disease-related metabolic pathways (Poliakov et al., 2014). However, when acquiring gene expression data, it often contains some inevitable noise, which causes errors in the calculation of conditional relatedness, well known as high false positive rate.

Another type of similarity, prior-knowledge similarity, is also used to measure gene–gene relatedness, based on the documented biological data and functional annotations in public domain, such as the Gene Ontology (GO) (Consortium, 2004), the KEGG (Kanehisa and Goto, 2000), the Reactome (de Bono et al., 2005), the OrthoDB (Zdobnov et al., 2016), the TRRUST (Han and Puri, 2018), *etc.* It brings high accuracy (ACC) (Diebold and Mariano, 1995), as the prior-knowledge similarity is confirmed by the biological experiment. But the biological experiment is usually conducted in a normal condition, meaning prior-knowledge similarity is hardly used for measuring conditional relatedness.

By the above understanding, integration of expression and prior-knowledge similarities is an effective way to avoid the shortage of using only one category of similarity to measuring conditional relatedness between genes, as a pair of genes with high expression similarity but low prior-knowledge similarity implies their relatedness is most likely a false prediction by co-expression analysis, and the two genes with low expression similarity but high prior-knowledge similarity implies their relatedness is not specific in the condition. The gene pair with both high expression and prior-knowledge similarities should be scored a high rank and recommended by a model. Wang et al. proposed a support vector machine (SVM) model using both expression and prior-knowledge similarities to measure conditional relatedness between a pair of genes, and their computational results showed the proposed model outperforms the existing co-expression analysis methods and other integration models (Wang et al., 2019). The combination of both kinds of similarities has been also succeeded in other related biological issues, *e.g.*, detection of protein–protein interaction (PPI) (Jing and Ng, 2010), measuring functional similarity of gene products (Mistry and Pavlidis, 2008), and identification of disease-causing gene (Mohammadi et al., 2011).

Because of the fast growth of the deep learning technology, deep learning algorithms have outperformed the state-of-the-art traditional machine learning algorithms in many research field of bioinformatics. Babak et al. adapted the deep learning convolutional neural network to the task of predicting sequence specificities and showed that they compete favorably with the state of the art (Babak et al., 2015), and their results show that their approach outperforms other state-of-the-art methods. Pan and Shen proposed a deep learning-based framework by using a novel hybrid convolutional neural network and deep belief network to predict the RNA-binding proteins (RBP) interaction sites and motifs on RNAs.They validate their method on 31 large-scale datasets, and their experiments show that the average area under the curve (AUC) (Lobo et al., 2010) can be improved by 8% compared to the best single-source-based predictor (Pan and Shen, 2017). Trebeschi et al. applied the deep learning methods to automatic localization and segmentation of rectal cancers on multiparametric MRI, and their results demonstrate that deep learning can perform accurate localization and segmentation of rectal cancer in multiparametric MRI in the majority of patients (Trebeschi et al., 2017). Gao et al. proposed a new computational approach based on deep neural networks to predict tRNA gene sequences, and their proposed methods outperformed the existing methods under different metrics (Gao et al., 2019).

Motivated by the above mentioned, we develop a novel deep learning model of convolutional neural network (CNN) with a fully connected first layer, named fully convolutional neural network (FCNN), to measure conditional relatedness between genes using both expression and prior-knowledge similarities. The goal of our model is to keep and recommend gene pairs with both high expression and prior-knowledge similarities. The fully connected first layer makes our model extracting more useful information than traditional CNN and the rest CNN structure makes our model easier to train than all fully connected deep learning models. In line of the above two advantages and integrating of co-expression and prior-knowledge similarities, FCNN model calls better results than other models and methods for identifying gene–gene interactions and constructing cancer gene networks. First, the FCNN model acquires an average 3.0% and 2.7% higher ACC values on validation and test samples collected from the COXPRESdb, KEGG, and TRRUST databases and a benchmark dataset of Xiao-Yong et al. research (Xiao-Yong et al., 2010). Then we perform a further verification on the samples from the GeneFriends and DIP databases, and the FCNN model obtains an average of 1.8% and 7.6% higher accuracy, respectively. Finally, the FCNN model is utilized to construct cancer gene networks, which also obtains more practical results, comparing with other models and methods. The source code of FCNN, as well as the datasets and results of this research, are freely available in https://bmbl.bmi.osumc.edu/FCNN. 

## Materials and Methods

### Dataset Collection

We take gene pairs with/without expression similarity (co-expression) and prior-knowledge similarity (protein–protein interaction, involvement in a same pathway, and transcriptional regulation) as samples to compose a whole dataset to make our model be trained to predict gene pairs with high expression similarity as well as those with high prior-knowledge similarity at the same time, *i.e.*, to identify gene pairs with both high expression and prior-knowledge similarities. Therefore, the dataset used for training, validation, and test consists of two sub-datasets, so called co-expression sub-dataset and prior-knowledge sub-dataset.

The co-expression sub-dataset is collected from the COXPRESdb database (Release v7.1) (Yasunobu et al., 2015), where co-expressed gene pairs are sorted ascendingly by the mutual rank (MR) (Obayashi and Kinoshita, 2009). The smaller the MR value is, the higher co-expression it has. For each gene, we select the top 30 co-expressed genes to compose 30 co-expressed gene pairs from the Hsa-u.v18-10 and Mmu-u.v18-10 datasets in the COXPRESdb database, respectively. Then we select gene pairs as positive samples that they are commonly co-expressed in Hsa-u.v18-10 and Mmu-u.v18-10 datasets. To relieve the imbalanced problem between positive and negative samples, for each gene, we select middle 60 non-co-expressed genes to compose negative samples, similarly as composing the positives, where negative samples are the non-co-expressed gene pairs with PPC values around 0. There are 32,735 positive samples and 26,782 negative samples in the sub-dataset.

The prior-knowledge sub-dataset is composed of three parts. A) We collect gene-pair samples from the KEGG database (Release Nov 1, 2018) (Kanehisa, 2002) as the first part, where positive samples are gene pairs composited by the genes involved in at least two same pathways, and the negative samples are randomly selected gene pairs composited by the genes never engaged in the same pathway, with the same number of the positives. There are 11,526 positive samples and 11,526 negative samples in the first part. B) Next, for the second part, we use 15,222 gene pairs with PPI from a benchmark dataset of Pan et al. research (Pan et al., 2010) as the positive samples and 21,579 gene pairs without PPI as the negatives. C) In terms of the third part of the sub-dataset, we collect 7,361 gene pairs with the transcriptional regulation records in the TRRUST database (Release v2) (Han et al., 2017) as the positive samples and 7,361 gene pairs by random permutation of the transcription factor and the regulated gene in the positive ones (Nakamura et al., 2004; De et al., 2005; Wang et al., 2019).

Finally, there are a total of 66,844 positive and 67,248 negative samples. Specially, some negative samples were obtained by permutation of the positives and were then selected randomly to ensure the same number of positives for construction of a model with high generalization. And to avoid the bias of random permutation and selection of negative samples, we conduct the above process 100 times, rising to 100 datasets, in each of which a fixed percentage of the samples are used to training, validation, and test, according to the detailed proportion of the sub and sub-sub datasets. Also, the labels for the positive gene pairs are marked as 1s and those for the negatives as 0s. The details of each sub-dataset are showcased in **Table 1**.

**Table 1:** The structure of FCNN dataset.

Sub dataset	Sub-sub dataset	Type of gene pair	Sample size
Co-expression	Co-expression	Positive	32,735
		Negative	26,782
Prior-knowledge	KEGG	Positive	11,526
		Negative	11,526
	PPI	Positive	15,222
		Negative	21,579
	TRRUST	Positive	7,136
		Negative	7,136
DIP	DIP	Positive	1,396
		Negative	1,396
GeneFriends	GeneFriends	Positive	8,675
		Negative	8,675

For model verification, the gene pairs downloaded from the GeneFriends (Release v3.1) (Sipko et al., 2015) and DIP (Release Feb 13, 2017) (Xenarios et al.) databases are utilized as samples. In the GeneFriends database, we select overall 8,675 co-expressed gene pairs with top 20 PCC values for each gene as the positive samples. Because there is only a small part of genes that are co-expressed in the human genome, we used 8,675 gene pairs obtained by random permutation of the first and second genes in the positive gene pairs as the negative samples. Similarly, 1,396 gene pairs with direct PPI collected from the DIP database are used as the positive samples. Considering gene pairs with real PPIs are rare in the whole human genome, the 1,396 gene pairs by permutation of the two genes in the positive samples are used as the negatives. To avoid the bias of permutation, we conduct the above process 100 times, rising to 100 datasets from the GeneFriends and DIP databases, respectively.

### Gene-Pair Features Calculation

To measure conditional relatedness between a pair of genes and avoid the deficiencies of using a single type of feature, we use two kinds of features of gene pairs, including the expression similarities and prior-knowledge similarities.

In the former one, there are seven features, which are the average expression level of each gene of a gene pair, including *Mean* *_1_* and *Mean* *_2_*, and five co-expression levels, including *PCC*, *SRC*, *PPC*, *MI*, and *CMI*. The expression data for calculation of expression similarities are collected from the GEO datasets (Barrett et al., 2012) based on the Affymetrix Human Genome U133 Plus 2.0 Array platform (released on Nov 2003). Then a pre-processing is executed, including log2 scale and quantile normalization.

The latter one contains five features such as GO similarity (*GOsim*) (Wang et al., 2007), subcellular location similarity (*LCsim*) (Yu et al., 2010), hormonology similarity (*HGsim*) (Chen and Vitkup, 2006), Reactome similarity (*RCsim*) (David et al., 2014), and transcriptional regulation similarity (*FRsim*) (Nagafuchi et al., 1991). The details of these features are defined as follows.

(1) $$GOsim_{i,j}=\max_{g\in G_{i,}q\in G_{j,}}\frac{\log(Pms{\left(g,q\right)}^{2})}{\log(P(g)+\log P(q))}$$

where *G* *_i_* and *G* *_j_* represent the GO term sets used for annotating gene *i* and *j*, respectively; *p(g)* represents the probability of a gene annotated by an instance of GO term *g*, and *Pms(g,q)* represents the minimum probability of a gene annotated by an instance of a common ancestor GO term of *g* and *q*. The GO terms of genes used here are the biological process GO terms with experimental evidence downloaded from the GO database (Kumari et al., 2012), where a GO tree is built by the relations among GO terms, including “is a”, “part of”, “has part”, and “regulates”.

(2) $$LCsim_{i,j}=\frac{\left|S_{i}\cap S_{j}\right|}{\left|S_{i}\cup S_{j}\right|}$$

where *S* *_i_* and *S* *_j_* represent the subcellular sets of two proteins encoded by gene *i* and gene *j*, respectively. The subcellular information of genes is collected from the GO database.

(3) $$HGsim_{i,j}=\frac{L\times K-v_{i}\times v_{j}}{\sqrt{\left(L\times v_{i}-v_{i}^{2})(L\times v_{j}-v_{j}^{2}\right)}}$$

where *v* *_i_* and *v* *_j_* represent the number of species whose genome contains homologous genes of gene *i* and *j*, respectively; *L* represents the total number of species; and *K* represents the number of species whose genome contains the homologous gene of both gene *i* and *j*.

(4) $$RCsim_{i,j}=1-\frac{d_{i,j}}{d_{\text{max}}}$$

where *d* *_i,j_* represents the shortest distance between gene *i* and gene *j* in the graph constructed by gene–gene interactions collected from the Reactome database (Croft et al., 2011), and *d* *_max_* represents the shortest distance of the farthest gene pair.

(5) $$FRsim_{i,j}=\{\begin{array}{c} 1,\text{if there is a transcriptional regulation record}\\ 0,\text{ otherwise} \end{array}$$

where *FRsim* *_i,j_* is equal to 1, if there is a transcriptional regulation between gene *i* and *j* recorded in the HTRIdb database (Bovolenta et al., 2012), and is equal to 0, otherwise. Meanwhile, all the databases and relevant data source used to compute these two kinds of gene-pair features are listed in **Supplementary Table S5**.

### Model Construction

**Figure 1:**
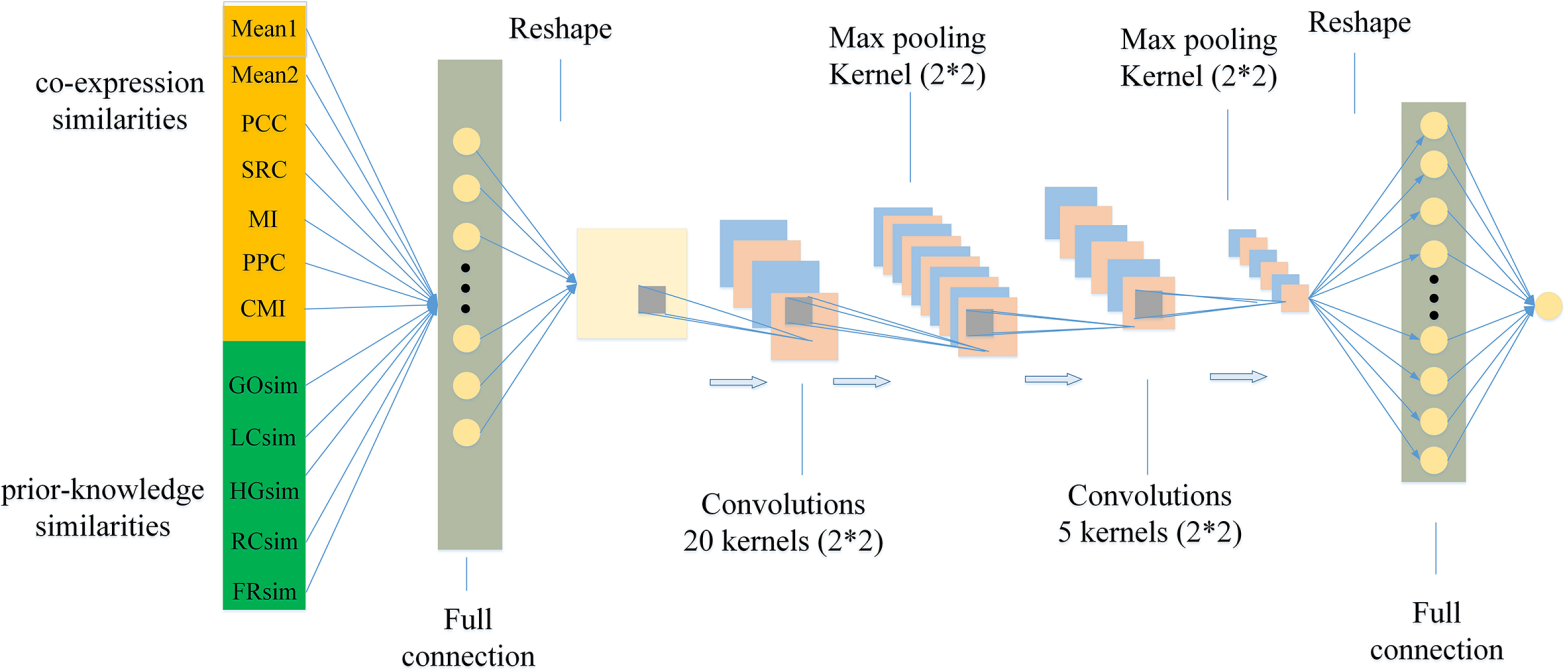
The structure of the FCNN model.

In the study, we design a model using CNN with a fully connected first layer, named FCNN to measure conditional relatedness of gene pairs shown as **Figure 1**. On one hand, the fully connected first layer of FCNN keeps our model from ignoring important feature combination. On the other hand, the CNN structure makes our model easy to train because of its parameter sharing and sparse connections. In detail, the model contains six layers. The first layer is a fully connected layer with 81 neurons and used for getting as much information as possible. The 12 features *X* = [*x*_1_,…,*x*_12_] as the inputs are fed into this layer to get the activation score a*_j_* of neural *j*:

(6) $$a_{j}=\sum_{i=1}^{12}\omega_{i,j}\;x_{i}+b_{j}$$

where ω*_ij_* represents the weight between the *x* *_i_* and neural *j*; and *b* *_j_* represents the bias. Then we reshape the output *A*_1_ = [*a*_1_,*a*_2_,…,*a*_81_] into a 9*9 matrix $A_{1}^{\prime }$:

(7) $$A_{1}^{\prime }=\left[\begin{array}{ccc} a_{1} & \cdots & a_{9}\\ \;\vdots & O & \;\vdots \\ a_{73} & \cdots & a_{81} \end{array}\right]$$

which is convenient to operate the convolution. The second layer is a convolutional layer using 20 convolutional kernels of size 2*2 and stride of 1. The output of each neuron of this layer is the convolution between a kernel matrix and a part of the input. The result *A*_2_ of the second layer is defined as:

(8) $$A_{2}=tanh(Conv(A_{1}^{\prime }))$$

where *Conv*(⋅) represents the convolution operation and *ReLU*(⋅) represents the rectified linear unit function. The third layer is a maximum pooling layer with the kernel of size 2*2 and stride of 2, which is used to down sample and reduce the dim of input by selecting the maximum value in each input. The output from the maximum pooling is recorded as *A*_3_:

(9) $$A_{3}=Max\_pool(A_{2})$$

A dropout operation is used on the third layer to randomly reduce a part of its output to avoid overfitting. The fourth layer is a convolutional layer with five kernels, and its kernel size is 2*2 with stride 1. The fifth layer is a maximum pooling layer with the kernel of size 2*2 and stride of 2. The purpose of using these layers is to further extract the information of the input features and improve the accuracy of the prediction. The results *A*_4_ and *A*_5_ of the fourth and the fifth layers are defined as

(10) $$A_{4}=tanh(Conv(A_{3}))$$

(11) $$A_{5}=Max\_pool(A_{4})$$

where *tanh(.)* represents the hyperbolic tangent activation function. The last layer is a fully connected output layer with the predicted conditional relatedness ${\hat{y}}_{k}$ of sample *k* defined as

(12) $${\hat{y}}_{k}=Sigmoid(W_{f}^{T}\cdot A{'}_{5}+b_{f})$$

(13) $$Sigmoid(x)=\frac{1}{1+e^{-x}}$$

where $A_{5}^{\prime }$ represents the reshaped vector of *A*_5_; *W* *_f_* and *b* *_f_* represent the weight vector and the bias of the final layer, respectively. We apply the Binary Cross Entropy loss (BCEloss) as the loss function of FCNN model defined as

(14) $$BCEloss=-[y_{k}\log({\hat{y}}_{k})+(1-y_{k})\log(1-{\hat{y}}_{k})]$$

where *y* *_k_* represents ground true label 1/0 of the positive/negative sample *k*, and *K* represents the total number of all samples. The optimal algorithm is RMSPROP (Zhang et al., 2019).

Based on the CNN structure with a fully connected first layer, our model is trained by grid-search 10-fold cross-validation, and the hyper-parameters with the highest AUC value of the whole cross-validation are employed, including kernel size, stride, *etc.* For the detailed description of the architecture and hyper-parameters, see Optimizing the FCNN Model section.

### Experimental Design

**Figure 2:**
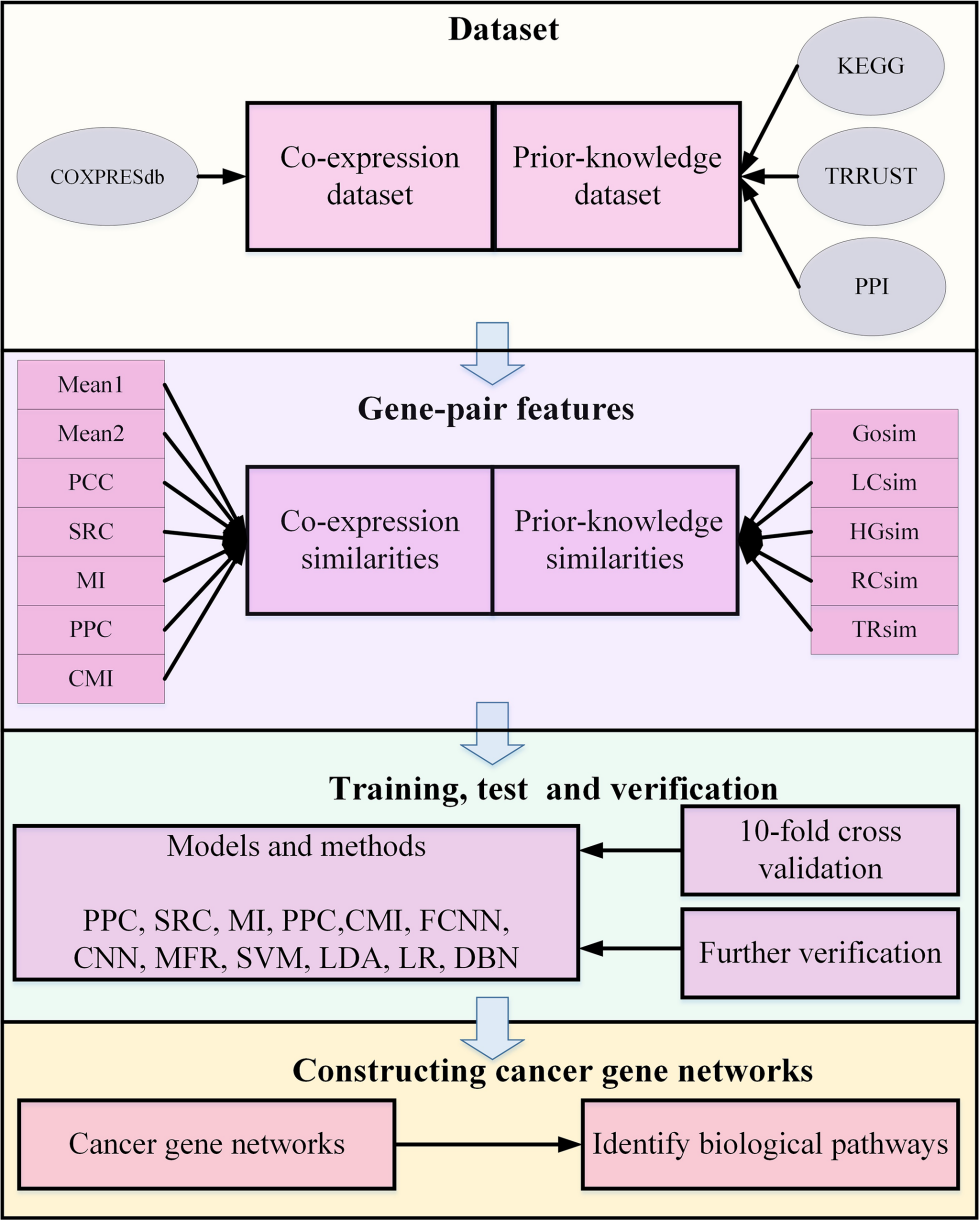
The flowchart of experimental design in biological pathways identification.

Herein, our experiment breaks down four parts, depicted as **Figure 2**, in detail. First, gene-pair samples are collected from three databases and a benchmark dataset to compose the dataset for FCNN construction, which contains co-expression and prior-knowledge sub-datasets. Second, 12 gene-pair features are calculated, including seven expression similarities and five prior-knowledge similarities. Third, FCNN is constructed by grid search in a 10-fold cross-validation process. Finally, FCNN is evaluated by comparing with 12 models and methods in 10-fold cross-validation, test, verification, and construction of gene network.

The 12 compared models and methods consist by seven models, including logistic regression (LR), linear discriminant analysis (LDA), SVM, deep belief network (DBN), fully connected neural network (FNN), CNN, and MFR (Wang et al., 2019), as well as five co-expression analysis methods, including PCC, SRC, MI, PPC, and CMI. In these models and methods, LR, LDA, and SVM are traditional machine learning technologies applied in many fields (Zhang et al., 2006; AndrewCucchiara, 2012; Asafu-Adjei et al., 2013). 

Specifically, the SVM model is constructed with the radial basis kernel function. DBN is a classical deep learning generation model, which combines restricted Boltzmann machine (Pang et al., 2018) and neural network structure. Multi-Features Relatedness (MFR) is a SVM-based model with linear kernel function proposed recently, integrating both expression and prior-knowledge similarities to measuring conditional relatedness. And PCC, SRC, MI, PPC, and CMI are traditional methods for measuring conditional relatedness between a pair of genes.

For each model and method, we conduct 10-fold cross-validation using 81% samples in dataset collected from the COXPRESdb, KEGG, and TRRUST databases and a benchmark dataset of Pan et al. research (Pan et al., 2010) for training, 9% samples for validation, and the rest 10% for test. And the results of validation and test are used to compare models and methods in terms of precision. Moreover, samples obtained from the GeneFriends and DIP databases are used for further verification to compare different models or methods in robustness. We also compare the practicability of models and methods in terms of cancer gene network construction. To compare the performance of each model or method, we select the receiver operating characteristic curve (ROC) with its AUC (Lobo et al., 2010) and the ACC value as the criteria.

## Results

### Optimizing the FCNN Model

We built our parameterized FCNN model using Pytorch (Aorte et al., 2019). The optimal hyper-parameters are obtained from various combinations based on baseline parameters by grid search within 10-fold cross-validation. We test hyper-parameter combinations containing the kernel size, stride, learning rate, activation functions, dropout probability, *etc.*, and get the experimental results of the different hyper-parameters shown as **Table 2**. Specially, the FCNN model is trained by minimizing the BCEloss with RMSprop optimizer (Zhao et al., 2019) in the light of the AUC of validation and test datasets. As shown in **Table 2**, the best hyper-parameters for combination of activation function, the kernel size, stride, the number of neurons in the first layer, learning rate, the dropout probability, and the batch size is Tanh_Tanh, 2, 1, 81, 0.001, 0.1, and 250, respectively.

**Table 2:** Effects of the varied hyper-parameters through a 10-fold cross-validation in terms of AUC based on the validation and test datasets.

Hyper-parameter	Parameter	Validation	Test
Kernel size	2	**0.8310**	**0.8321**
	3	0.8121	0.8172
Stride	1	**0.8310**	**0.8321**
	2	0.8089	0.8156
Number of neurons	25	0.8191	0.8232
	81	**0.8310**	**0.8321**
	169	0.8189	0.8236
Learning rate	0.01	0.8250	0.8296
	0.001	**0.8310**	**0.8321**
	0.0001	0.7763	0.7802
Dropout probability	0.1	**0.8310**	**0.8321**
	0.2	0.8196	0.8228
	0.3	0.8180	0.8227
Batch size	200	0.8166	0.8231
	250	**0.8310**	**0.8321**
	300	0.8135	0.8209
Activation function	ReLU_ReLU	0.8132	0.8224
	ReLU_Sigmoid	0.8127	0.8210
	ReLU_Tanh	0.8127	0.8242
	Sigmoid_ReLU	0.8224	0.8296
	Sigmoid_Sigmoid	0.8245	0.8301
	Sigmoid_Tanh	0.8271	0.8308
	Tanh_ReLU	0.8253	0.8297
	Tanh_Sigmoid	0.8245	0.8309
	Tanh_Tanh	**0.8310**	**0.8321**

FCNN model obtains the optimal AUC value, based on the different hyper-parameters combinations.

**Table 2** reflects the experimental results of the combining hyper-parameters. The nine kinds of combination of three activation functions (ReLU, Sigmoid, and Tanh) are evaluated. As a result, combination of Tanh and Tanh is optimal. The kernel size and the stride of the FCNN model are changed to 2 and 3, and 1 and 2, respectively. The kernel of 2 and the stride of 1 are the best suitable for our approach, respectively. The neuron number of the first layer is changed to 5*5, 9*9, and 13*13, and we find 9*9 is optimal. The learning rate is changed to 0.0001, 0.001, and 0.01, and the learning rate of 0.001 shows our approach obtains the best performance in both validation and test AUC. To avoid the overfitting, the dropout probability is applied in our approach, changed to 0.1, 0.2, and 0.3. The dropout probability of 0.1 presents the highest AUC in training and test; meanwhile, the larger the dropout probability, the lower the AUCs on validate and test datasets. And then the batch size for the model is also changed to 200, 250, and 300, which shows that the batch size of 250 gets the best performance. To sum up, the combination of the kernel size of 2, the stride of 1, the neuron number of 81 in the first layer, the learning rate of 0.01, the dropout probability of 0.1, and the batch size of 250 is optimal. And we also list the optimal condition under a single hyper-parameter, based on our experiments.

### Comparison With Existing Methods

**Figure 3:**
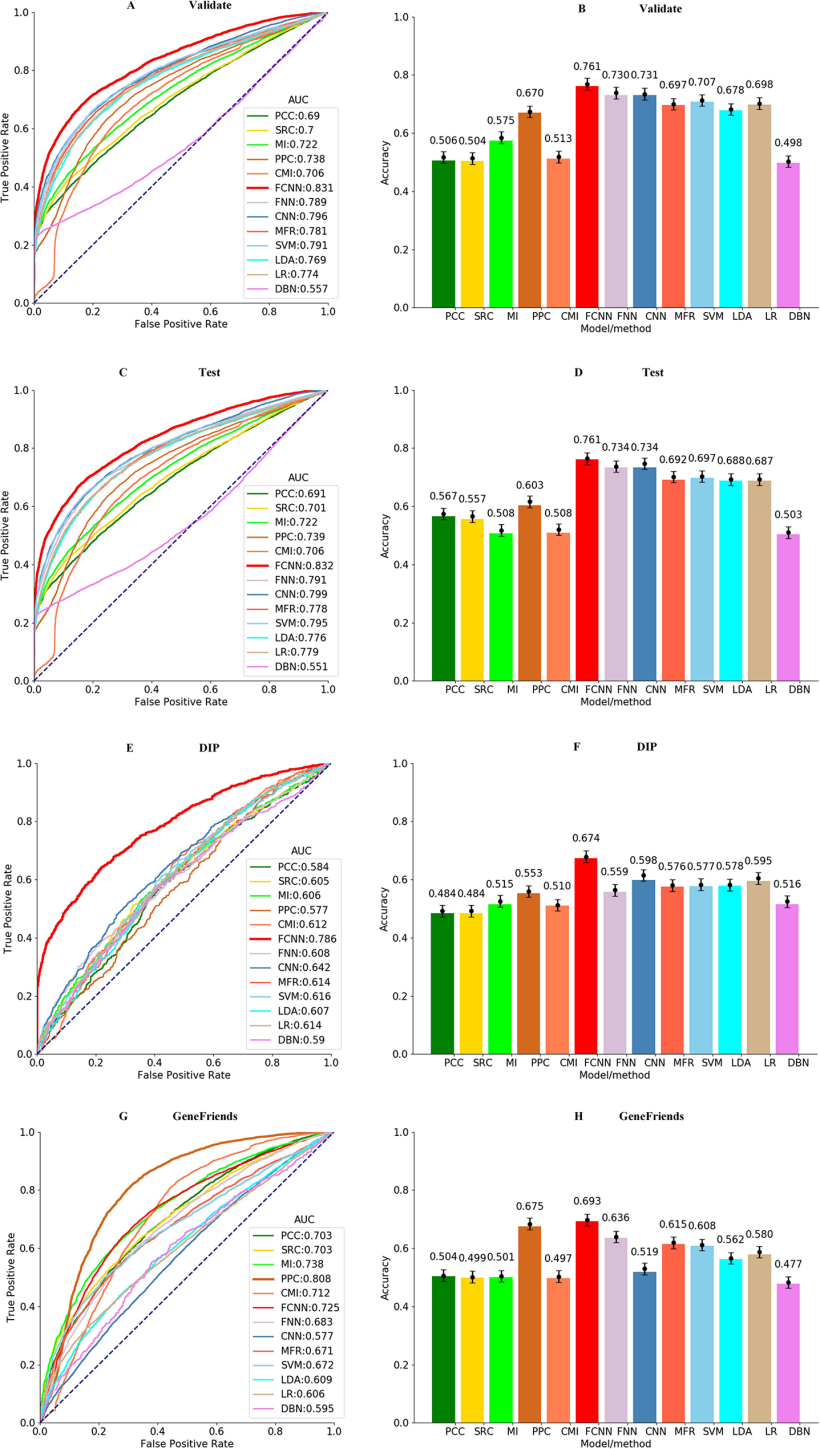
ROCs of all models and methods for identifying gene–gene interactions in the **(A)** validation, **(C)** test, **(E)** DIP, and **(G)** GeneFriends datasets. ACCs of all models and methods for identifying gene–gene interactions in the **(B)** validation, **(D)** test, **(F)** DIP, and **(H)** GeneFriends datasets.

The best parameters of all models are obtained by grid search within 10-fold cross-validation, and the results of the final models with the best parameters are applied to compare models and methods in terms of precision. As shown in **Figures 3A, B**, most machine learning models perform better than the co-expression analysis methods, and our FCNN model has the highest AUC value of 0.831 and ACC of 0.761 than the others. CNN model is better than others except for the FCNN model, with an AUC value of 0.796 and ACC of 0.731, but the DBN model performs the worst among all models and methods. In the light of **Figures 3C–D**, the FCNN model obtains the highest AUC and ACC against all models and methods on the test dataset. And the CNN model yields higher AUC value of 0.799 and ACC value of 0.734, which is better than other models and methods besides the FCNN model.

To test the generalization and robustness of all models and methods on the samples obtained from the GeneFriends and DIP databases, all models applied on this further verification are trained without samples from the GeneFriends and DIP databases. As shown in **Figures 3E–H**, the result on the GeneFriends database reflects the robustness of models and methods in detecting gene–gene interactions from co-expression dataset, and the performance on the DIP database indicates generalization in identifying gene–gene interactions from the prior-knowledge datasets. **Figures 3E–H** shows that FCNN model obtains the third largest AUC value of 0.725 and the highest ACC value of 0.693 among all models and methods on the GeneFriends samples, and AUC and ACC values of FCNN model are better than others on the DIP samples, which are 0.786 and 0.674, respectively.

To clarify the performance of the trained FCNN model on the co-expression, PPI, KEGG, and TRRUST sub-sub datasets, respectively, we applied all models and methods to these four sub-sub datasets and the results shown as **Figure S1**. According to **Figure S1**, our approach achieves the highest AUC of 0.938, 0.578, and 0.532 on the co-expression, PPI, and TRRUST datasets, respectively. For the KEGG dataset, AUC of 0.628 of the FCNN model is a little lower than AUC of 0.63 the CNN model obtained. In light of the above results, it is reasonable that the AUC of FCNN model on the co-expression dataset is higher than on the prior-knowledge dataset, which reflects that our models identify the relationship of genes mainly depending on the co-expression information. And the prior-knowledge information only acts as an auxiliary role in the process of identifying gene relationships. To the best of our knowledge, the co-expression information can powerfully reflect the relatedness of genes in a real cell environment, but possibly contains some error messages. And the prior-knowledge information is invested to relieve these error messages, as the relatedness of gene pairs support by the prior-knowledge messages is global, meaning only a small part of those relatedness is activated on a certain condition. Meanwhile, it also implies our model is not suitable for catching the global relatedness of gene pairs support by the prior knowledge.

### Constructing Cancer Gene Networks

Genes act as a vital role in many human diseases, most of which often work with each other and affect human health (Li et al., 2018), and the weighed gene network provides an effective method to study the relationship between genes (Yang et al., 2014). There is a property of gene networks in which the genes involved in related biological processes are connected to each other to compose gene subnetworks with density inside connections and sparse outside connections, *i.e.*, genes in a module should be involved in related biological processes (Matteo et al., 2012). Here, the purpose of measuring conditional relatedness between genes is to detect the probability of these genes jointly involving in a biological process. Therefore, the better conditional relatedness is measured by a model for constructing gene network, the more distinctive such property is. Inspired by the above, we use this property to compare each model or method in the construction of gene networks. The conditional relatedness in this research is utilized to construct cancer gene networks, where nodes indicate genes and weights on edges indicate relatedness. The criterion is the number of metabolic pathways predicted significantly influenced by increased serine metabolism in cancers. We choose reprogramming serine metabolism as it is one of the hallmarks of cancer (Yang and Vousden, 2016). It is reported that serine metabolism is increased in various cancers, especially in bladder cancer (Massari et al., 2016), breast cancer (Locasale et al., 2011; Richard et al., 2011; Kim et al., 2014), colon cancer (Duthie, 2011; Jie et al., 2015; Yoon et al., 2015), and lung cancer (Piskac-Collier et al., 2011; Denicola et al., 2015), and supports several metabolic processes that are crucial for the growth and survival of cancer cells, such as DNA/RNA methylation (Maddocks et al., 2016), glutathione biosynthesis (Amelio et al., 2014), one-carbon metabolism (Yang and Vousden, 2016), *etc.* We conduct enrichment analysis on gene modules identified to be influenced by increased serine metabolism against all the pathways in the KEGG database and obtain significant enriched metabolic pathways (q-value < 0.01) (Storey, 2003). Then we count the number of how many of the significant enriched metabolic pathways are the ones reported to be related to enhanced serine metabolism in cancer tissues. The number shows how well the genes in a module are involved in related biological processes and reflects how well the conditional relatedness is measured by different models for gene network construction.

**Figure 4:**
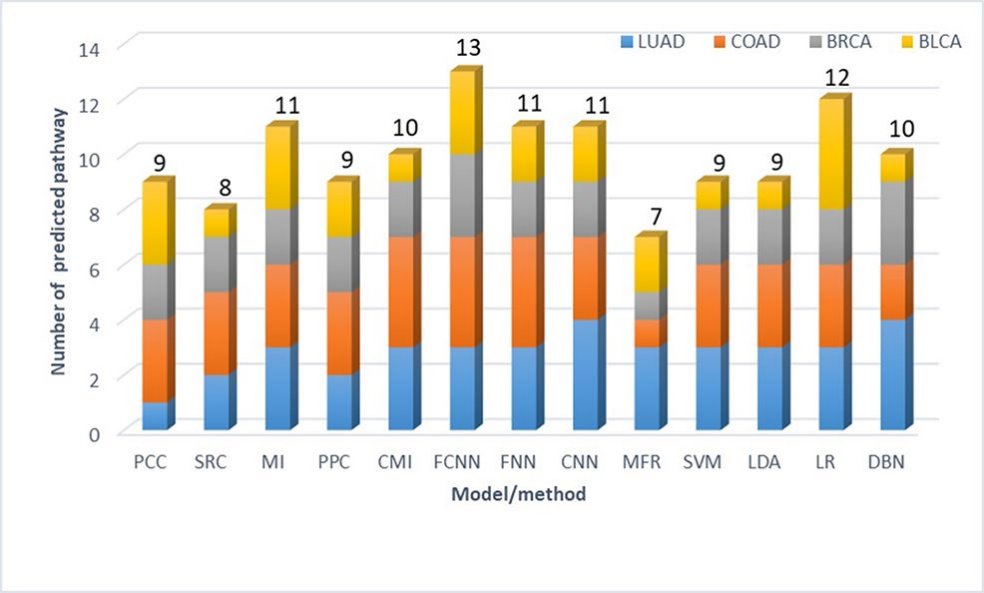
Number of metabolic pathways predicted to be directly influenced by increased serine metabolism in four cancer types.

First, we collect RNA-Seq gene expression data of four cancer types, including bladder urothelial carcinoma (BLCA), breast invasive carcinoma (BRCA), colon adenocarcinoma (COAD), and lung adenocarcinoma (LUAD) from the TCGA database (Hampton, 2006), the details of which are shown in **Table 3**. Second, up-regulated genes are identified using Limma t-test (Ritchie et al., 2015), with the fold-change of expression level in cancer *versus* normal tissue > 1.5 and P value < 0.05. Then the relatedness of each pair of up-regulated genes is calculated by FCNN model and 12 other models and methods. Especially, co-expression similarities used as features for each model are calculated using gene expression data in cancers. Third, we construct cancer gene networks, where nodes indicate up-regulated genes, and for each node, we link other nodes with the top 5 relatedness. There are a total of 13*4 gene networks for 13 models and methods in four cancer types. Fourth, we collect 11 enzyme-encoding genes that catalyze biological reactions of serine as the markers for serine metabolism, including *CBS*, *CBSL*, *PTDSS1*, *PTDSS2*, *SDS*, *SDSL*, *SHMT1*, *SHMT2*, *SPTLC1*, *SPTLC2*, *SPTLC3*, and *SRR*. The modules in each network are identified by fast modularity optimization algorithm (Zhang et al., 2009). And the modules with gene markers are defined as modules influenced by increased serine metabolism. We implement gene set enrichment analysis against KEGG pathways on such modules (Christina et al., 2007), by using the hyper-geometric test, with q-value < 0.01. Finally, the metabolic pathways confirmed to be significantly influenced by enhanced serine metabolism in cancer tissues are obtained by intersecting-enriched pathways with the ground truth (see **Supplement Tables 1–4**). As shown in **Figure 4**, we detect 13 significantly influenced pathways in FCNN-based gene network in four cancer types, which is the most among all models and methods.

**Table 3:** The number of samples in cancer and normal tissue.

Caner type	Samples in normal tissue	Samples in cancer
LUAD	515	19
COAD	285	113
BRCA	1095	41
BLCA	408	59

## Discussion

Recent advances in deep learning and bioinformatics stimulate considerable interest in measuring the relatedness of genes, and such pursuit is necessary, which not only speeds up transition from machine learning methods based on measuring correlation to deep learning methods but also can reveal some potential relationship between genes.

Our approach integrates a fully connected layer and the CNN structure for measuring conditional relatedness between genes by integrating co-expression and prior-knowledge similarities. Meanwhile, we demonstrate that this approach is available and effective by experiments on different datasets. To verify our model, we compare the FCNN model with other seven models and five co-expression analysis methods in validation, test, and further verification. The results show that most of machine learning models have higher AUC and ACC values than co-expression analysis methods, implying a combination of both co-expression and prior-knowledge similarities has more obvious advantages in terms of measuring conditional relatedness than using only co-expression similarities. The FCNN model obtains the best performance among machine learning models, which proves deep-learning-based models can more effectively detect the complex map relations between similarities and conditional relatedness than traditional algorithms, such as FNN, MFR, LR, LDA, SVM, and so on. Especially, FCNN model successfully calls a better result than CNN model, which indicates the fully connected first layer persists in our model from ignoring useful combinations of features and the remaining CNN structure with parameter sharing and connection sparsity help our model to be easily trained on the medium-sized dataset. All the above advantages make FCNN model more practical, and as a result, it achieves the best performance in the construction of cancer gene networks. However, PPC and MI obtain higher AUC values on the GeneFriends samples than the FCNN model, mainly because the gene–gene interactions collected from the GeneFriends database are predicted by PCC, making PCC have a natural advantage comparing with other models or methods. And MI has some resemblance with PCC (Yan et al., 2019), which makes it gain the second best result on the GeneFriends dataset.

In line with the performance of the FCNN model, for the next step, we will collect more data, extract more features of gene pairs, and plan to optimize the structure of the model to improve the performance. Meanwhile, we generate some of the negative datasets by random permutation following the way of the references, which may suffer from issue of neglecting tissue specificity; therefore, we will improve this process in our coming researches. Moreover, deep learning is an extremely active research community that is garnering more and more focus from academia, and we expect that deep learning models like this hybrid architecture will be continually explored for the purpose of measuring the relatedness between genes.

## Conclusion

In conclusion, the FCNN model is a novel deep learning model of CNN with a fully connected first layer, combining co-expression and prior-knowledge similarities to measure conditional relatedness between genes. For benchmarking purposes, we compare the FCNN model to existing models and co-expression analysis methods; our proposed model obtains the best performance of identifying gene–gene interaction invalidation, test, and further verification. Meanwhile, we estimate the performance of all models and methods on the co-expression and prior-knowledge sub-datasets, respectively, which show that the FCNN model is optimal. In terms of constructing gene networks, the FCNN model also outperforms other compared models and methods and achieves more practical results.

## Data Availability Statement

The datasets and results of this study, and code of the FCNN model can be freely obtained from https://bmbl.bmi.osumc.edu/FCNN for academic uses and biological analysis.

## Author Contributions

SZ and YT collected the data and performed the experiments. YW conceived the project. YW and QM designed the study. YT, SZ, LY, and SY wrote the manuscript. All authors read and approved the final manuscript for publication.

## Funding

This research was funded by the National Natural Science Foundation of China (Nos. 61572227, 61872418) and the Development Project of Jilin Province of China (Nos. 20170203002GX, 20170520063JH, 20180414012GH, 20190201293JC).

## Conflict of Interest

The authors declare that the research was conducted in the absence of any commercial or financial relationships that could be construed as a potential conflict of interest.
