# Supplementary material for: Measurement of Conditional Relatedness Between Genes Using Fully Convolutional Neural Network
Source: Front Genet. 2019 Oct 22;10:1009. doi: 10.3389/fgene.2019.01009 (PMC6818468; doi:10.3389/fgene.2019.01009)
Supplement: Supplementary file 1 [file DataSheet_1.docx]

***Supplementary Material***

# Supplementary Tables

The enriched metabolic pathways (q-value < 0.01) confirmed as the ones significantly influenced by increased serine metabolism in cancers for different models and methods are shown as follows.

Table S1. Metabolic pathways are confirmed to be directly influenced by increased serine metabolism in the 13 COAD gene networks

| Model/Method | Pathway | q-value |
| --- | --- | --- |
| PCC | Cysteine and methionine metabolism | 5.92e-06 |
| PCC | Glutathione metabolism | 1.831e-08 |
| PCC | Purine metabolism | 5.498e-14 |
| SRC | Glutathione metabolism | 1.049e-08 |
| SRC | Cysteine and methionine metabolism | 0.233e-03 |
| SRC | Purine metabolism | 3.390e-16 |
| MI | Cysteine and methionine metabolism | 2.782e-07 |
| MI | Glutathione metabolism | 0. 674e-3 |
| MI | Purine metabolism | 8.352e-25 |
| PPC | Cysteine and methionine metabolism | 2.523e-07 |
| PPC | Glutathione metabolism | 5.998e-10 |
| PPC | Purine metabolism | 4.029e-16 |
| CMI | Glycine, serine and threonine metabolism | 0.320e-02 |
| CMI | Cysteine and methionine metabolism | 1.179e-08 |
| CMI | Glutathione metabolism | 3.379e-12 |
| CMI | Purine metabolism | 2.381e-30 |
| **FCNN** | Cysteine and methionine metabolism | 0.476e-02 |
| **FCNN** | Glutathione metabolism | 0.653e-03 |
| **FCNN** | Purine metabolism | 5.210e-12 |
| **FCNN** | One carbon pool by folate | 0.178e-02 |
| FNN | Glycine, serine and threonine metabolism | 0.671e-02 |
| FNN | Cysteine and methionine metabolism | 0.641e-02 |
| FNN | Purine metabolism | 1.426e-14 |
| FNN | One carbon pool by folate | 7.248e-05 |
| CNN | Glycine, serine and threonine metabolism | 0.292e-02 |
| CNN | Glutathione metabolism | 0.303e-02 |
| CNN | Purine metabolism | 3.836e-05 |
| MFR | Purine metabolism | 1.324e-12 |
| SVM | Cysteine and methionine metabolism | 0.247e-02 |
| SVM | Glutathione metabolism | 0.169e-03 |
| SVM | Purine metabolism | 1.36e-12 |
| LDA | Cysteine and methionine metabolism | 0.106e-02 |
| LDA | Glutathione metabolism | 0.129e-02 |
| LDA | Purine metabolism | 1.295e-07 |
| LR | Cysteine and methionine metabolism | 8.729e-06 |
| LR | Glutathione metabolism | 1.639e-05 |
| LR | Purine metabolism | 9.089e-07 |
| DBN | Glutathione metabolism | 0.72e-02 |
| DBN | Purine metabolism | 4.976e-22 |

Table S2. Metabolic pathways are confirmed to be directly influenced by increased serine metabolism in the 13 LUAD gene networks

| Model/Method | Pathway | q-value |
| --- | --- | --- |
| PCC | Glutathione metabolism | 0.704e-02 |
| SRC | Glycine, serine and threonine metabolism | 0.423e-02 |
| SRC | Cysteine and methionine metabolism | 0.442e-03 |
| MI | Glycine, serine and threonine metabolism | 1.458e-07 |
| MI | Cysteine and methionine metabolism | 5.509e-06 |
| MI | Purine metabolism | 0.770e-02 |
| PPC | Cysteine and methionine metabolism | 3.789e-05 |
| PPC | Purine metabolism | 0.671e-03 |
| CMI | Glycine, serine and threonine metabolism | 0.233e-03 |
| CMI | Cysteine and methionine metabolism | 1.258e-05 |
| CMI | Purine metabolism | 0.631e-02 |
| **FCNN** | Glycine, serine and threonine metabolism | 0.901e-02 |
| **FCNN** | Cysteine and methionine metabolism | 0.901e-02 |
| **FCNN** | Purine metabolism | 0.225e-03 |
| FNN | Glycine, serine and threonine metabolism | 0.713e-02 |
| FNN | Cysteine and methionine metabolism | 0.450e-03 |
| FNN | Purine metabolism | 0.350e-03 |
| CNN | Glycine, serine and threonine metabolism | 0.390e-02 |
| CNN | Cysteine and methionine metabolism | 0.390e-02 |
| CNN | Glutathione metabolism | 0.479e-02 |
| CNN | Purine metabolism | 1.388e-06 |
| MFR | Cysteine and methionine metabolism | 0.541e-02 |
| MFR | Glutathione metabolism | 2.815e-07 |
| MFR | Purine metabolism | 0.708e-02 |
| SVM | Glycine, serine and threonine metabolism | 0.493e-02 |
| SVM | Cysteine and methionine metabolism | 0.214e-03 |
| SVM | Purine metabolism | 0.758e-02 |
| LDA | Glycine, serine and threonine metabolism | 3.111e-06 |
| LDA | Cysteine and methionine metabolism | 7.546e-08 |
| LDA | Glutathione metabolism | 1.805e-07 |
| LR | Glycine, serine and threonine metabolism | 0.398e-02 |
| LR | Cysteine and methionine metabolism | 0.142e-03 |
| LR | Purine metabolism | 0.641e-03 |
| DBN | Glycine, serine and threonine metabolism | 0.349e-02 |
| DBN | Cysteine and methionine metabolism | 7.213e-10 |
| DBN | Glutathione metabolism | 0.306e-03 |
| DBN | Purine metabolism | 0.572e-03 |

Table S3. Metabolic pathways are confirmed to be directly influenced by increased serine metabolism in the 13 BRCA gene networks

| Model/Method | Pathway | q-value |
| --- | --- | --- |
| PCC | Glycine, serine and threonine metabolism | 0. 81e-03 |
| PCC | Purine metabolism | 1.497e-08 |
| SRC | Glycine, serine and threonine metabolism | 0.908e-03 |
| SRC | Cysteine and methionine metabolism | 0.908e-03 |
| MI | Glycine, serine and threonine metabolism | 0.785e-03 |
| MI | Cysteine and methionine metabolism | 0.785e-03 |
| PPC | Cysteine and methionine metabolism | 0.116e-02 |
| PPC | Purine metabolism | 8.494e-05 |
| CMI | Glycine, serine and threonine metabolism | 0.200e-03 |
| CMI | Cysteine and methionine metabolism | 2.815e-06 |
| **FCNN** | Glycine, serine and threonine metabolism | 0.358e-03 |
| **FCNN** | Cysteine and methionine metabolism | 1.645e-05 |
| **FCNN** | Purine metabolism | 1.645e-05 |
| FNN | Glycine, serine and threonine metabolism | 0.557e-03 |
| FNN | Cysteine and methionine metabolism | 0.557e-03 |
| CNN | Cysteine and methionine metabolism | 0.260e-03 |
| CNN | Purine metabolism | 0.684e-03 |
| MFR | Cysteine and methionine metabolism | 0.135e-03 |
| SVM | Glycine, serine and threonine metabolism | 0.520e-02 |
| SVM | Cysteine and methionine metabolism | 0.418e-03 |
| LDA | Glycine, serine and threonine metabolism | 0.497e-03 |
| LDA | Cysteine and methionine metabolism | 0.498e-03 |
| LR | Glycine, serine and threonine metabolism | 0.614e-02 |
| LR | Cysteine and methionine metabolism | 0.524e-03 |
| DBN | Glycine, serine and threonine metabolism | 0.985e-02 |
| DBN | Cysteine and methionine metabolism | 3.968e-05 |
| DBN | Purine metabolism | 2.633e-10 |

Table S4. Metabolic pathways are confirmed to be directly influenced by increased serine metabolism in the 13 BLCA gene networks

| Model/Method | Pathway | q-value |
| --- | --- | --- |
| PCC | Cysteine and methionine metabolism | 0.554e-02 |
| PCC | Glutathione metabolism | 1.717e-06 |
| PCC | Purine metabolism | 2.286e-05 |
| SRC | Glutathione metabolism | 0.117e-02 |
| MI | Glycine, serine and threonine metabolism | 0.747e-02 |
| MI | Glutathione metabolism | 0.109e-02 |
| MI | Purine metabolism | 6.469e-06 |
| PPC | Glycine, serine and threonine metabolism | 0.167e-02 |
| PPC | Cysteine and methionine metabolism | 0.159e-02 |
| CMI | Cysteine and methionine metabolism | 0.475e-03 |
| **FCNN** | Glycine, serine and threonine metabolism | 0.684e-02 |
| **FCNN** | Cysteine and methionine metabolism | 0.650e-02 |
| **FCNN** | Purine metabolism | 0.364e-02 |
| FNN | Cysteine and methionine metabolism | 0.108e-02 |
| FNN | Purine metabolism | 0.205e-03 |
| CNN | Glycine, serine and threonine metabolism | 0.853e-02 |
| CNN | Purine metabolism | 0.434e-03 |
| MFR | Cysteine and methionine metabolism | 0.111e-03 |
| MFR | Purine metabolism | 7.393e-07 |
| SVM | Purine metabolism | 1.174e-06 |
| LDA | Cysteine and methionine metabolism | 0.396e-02 |
| LR | Cysteine and methionine metabolism | 5.771e-06 |
| LR | Glutathione metabolism | 6.044e-07 |
| LR | Purine metabolism | 4.102e-14 |
| LR | One carbon pool by folate | 0.217e-02 |
| DBN | Cysteine and methionine metabolism | 0.163e-02 |

Table S5. Databases are utilized to compute gene-pairs features

| Number | Database | Link |
| --- | --- | --- |
|  | GEO | https://www.ncbi.nlm.nih.gov/gds |
|  | GO | http://geneontology.org/ |
|  | Reactome | https://reactome.org/ |
|  | HTRIdb | http://www.lbbc.ibb.unesp.br/htri |
