# Supplementary material for: Measurement of Conditional Relatedness Between Genes Using Fully Convolutional Neural Network
Source: Front Genet. 2019 Oct 22;10:1009. doi: 10.3389/fgene.2019.01009 (PMC6818468; doi:10.3389/fgene.2019.01009)
Supplement: Supplementary file 2 [file Image_1.pdf]

# *Supplementary Material*

## 1 Supplementary Figures

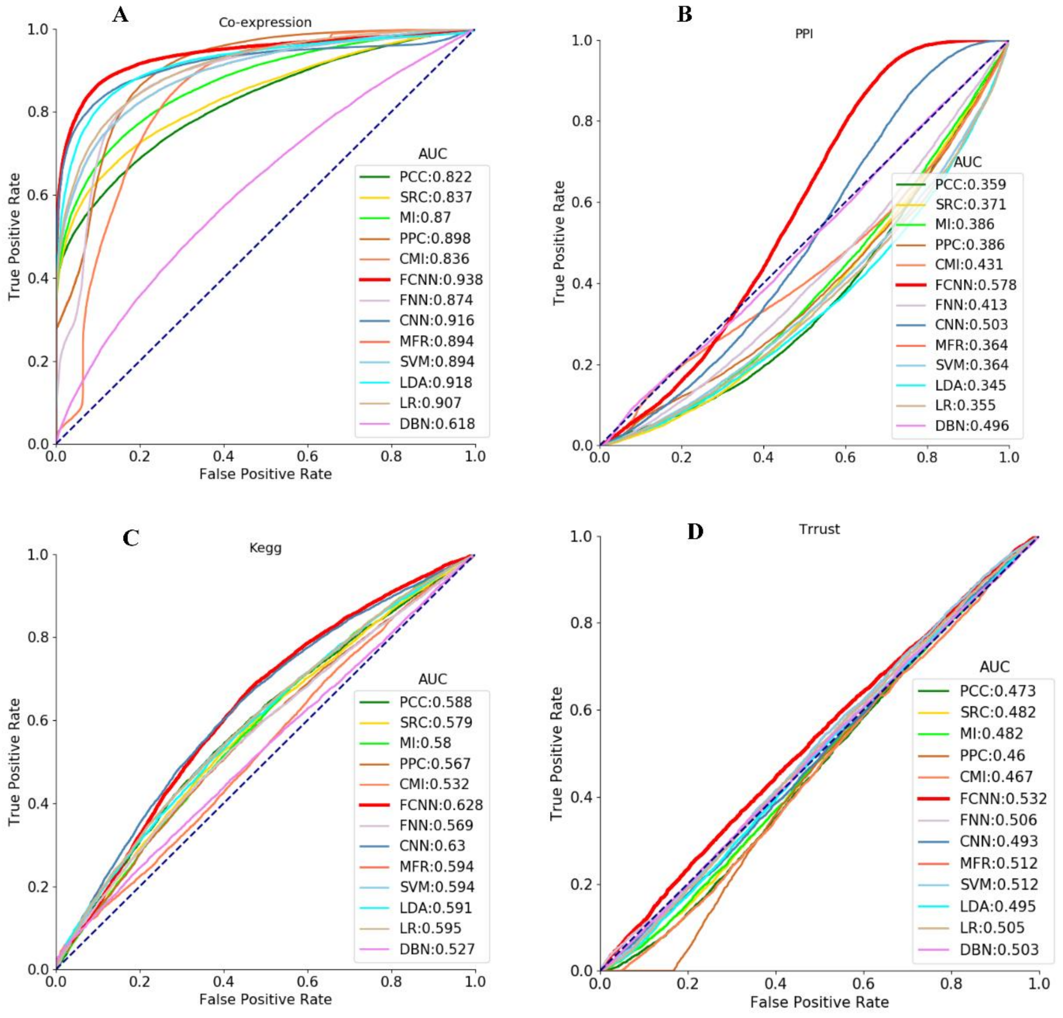


Figure S1. The ROCs of all models and methods on (A) co-expression, (B) PPI, (C) KEGG and (D) TRRUST sub-sub datasets.
